# Supplementary material for: Spectral Map for Slow Collective Variables, Markovian Dynamics, and Transition State Ensembles
Source: J Chem Theory Comput. 2024 Sep 12;20(18):7775–84. doi: 10.1021/acs.jctc.4c00428 (PMC11428138; doi:10.1021/acs.jctc.4c00428)
Supplement: Supplementary file 1 — ct4c00428_si_001.pdf [file ct4c00428_si_001.pdf]

Supporting Information:

Spectral Map for Slow Collective Variables, Markovian Dynamics, and Transition State Ensembles

Jakub Rydzewski*

Institute of Physics, Faculty of Physics, Astronomy and Informatics, Nicolaus Copernicus University, Grudziadzka 5, 87-100 Toruń, Poland

E-mail: jr@fizyka.umk.pl

Comment on the FiP35 Dataset

We performed our analysis on the first 100-μ trajectory from the FiP35 dataset, which was published in Ref. S1, instead of using a newer 1000-μ dataset provided by the same group in Ref. S2. We made this decision because we wanted to concentrate on the FiP35 miniprotein, which was more extensively discussed in the original work. It is important to note that, despite the difference in length, our results also match with the latter dataset, providing a solid foundation for our conclusions. Additionally, this also showcases the predictive capabilities of spectral map for learning slow conformational changes from shorter trajectories.

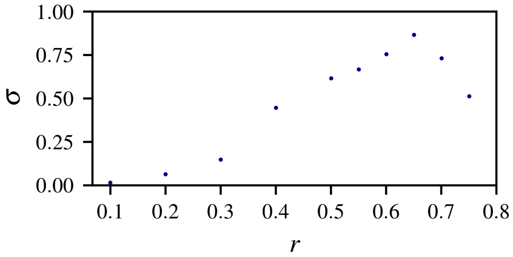

Figure S1: Maximized spectral gaps for different values of the fraction of neighborhood size used to estimate sample-dependent scale matrices.

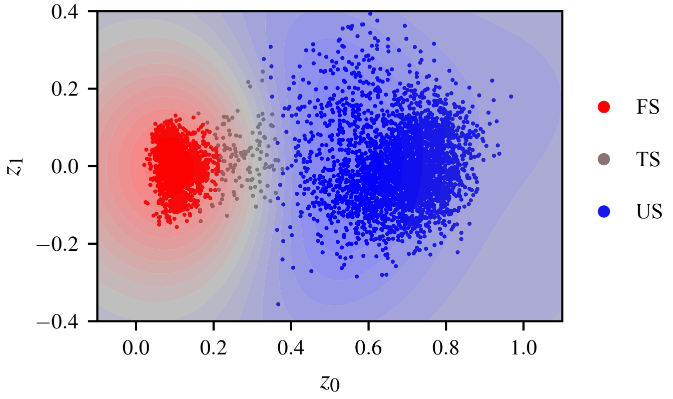

Figure S2: Support vector classifier trained based on kinetic partitioning of the reduced space performed by propagating Markov transition matrices from data batches. Decision boundaries are shown in red (folded), blue (unfolded), and gray (transition). The classifier is trained using a Gaussian kernel $\exp(-\|\mathbf{z}_k - \mathbf{z}_l\|^2/\epsilon)$ with a bandwidth ϵ of 0.1.

References

(S1) Shaw, D. E.; Maragakis, P.; Lindorff-Larsen, K.; Piana, S.; Dror, R. O.; Eastwood, M. P.; Bank, J. A.; Jumper, J. M.; Salmon, J. K.; Shan, Y. Atomic-Level Characterization of the Structural Dynamics of Proteins. *Science* **2010**, *330*, 341–346.

(S2) Lindorff-Larsen, K.; Piana, S.; Dror, R. O.; Shaw, D. E. How Fast-Folding Proteins Fold. *Science* **2011**, *334*, 517–520.

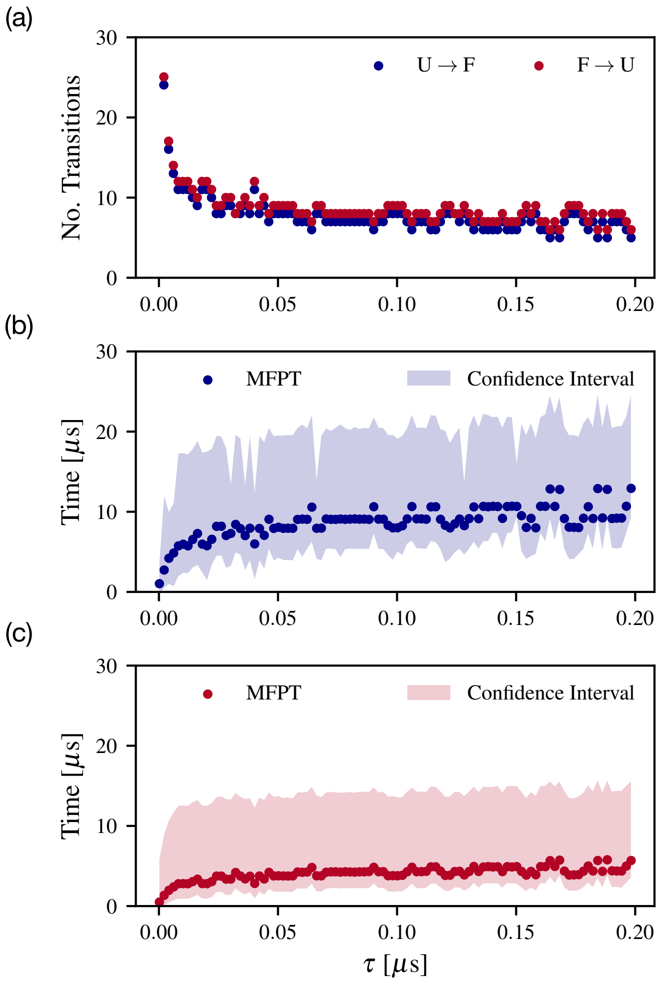

Figure S3: Mean first-passage times (MFPTs) for transitions between the folded and unfolded metastable states of FiP35 as a function of lag time τ. (a) Number of transitions between the states for the transition state ensemble determined by kinetic partitioning. (b) MFTPs for the transition from the folded to unfolded states and (c) for the reverse transition. Confidence intervals (0.95) are calculated using bootstrapping.

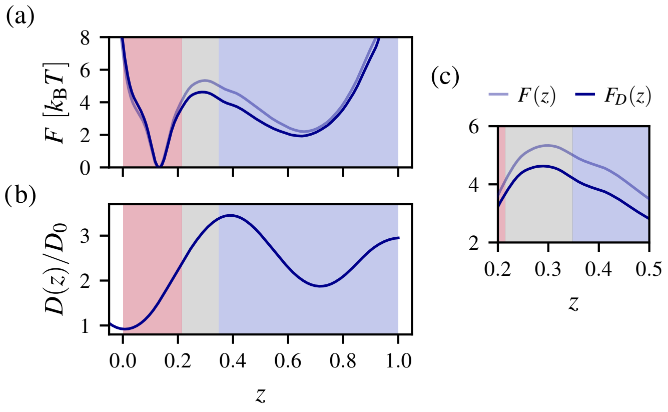

Figure S4: Effect of coordinate-dependent diffusion coefficients on the free-energy profile of FiP35. (a) Free-energy profiles: F and diffusion-dependent F_D. (b) Coordinate-dependent diffusion coefficients. (c) The most affected region of the free-energy profile from (a).

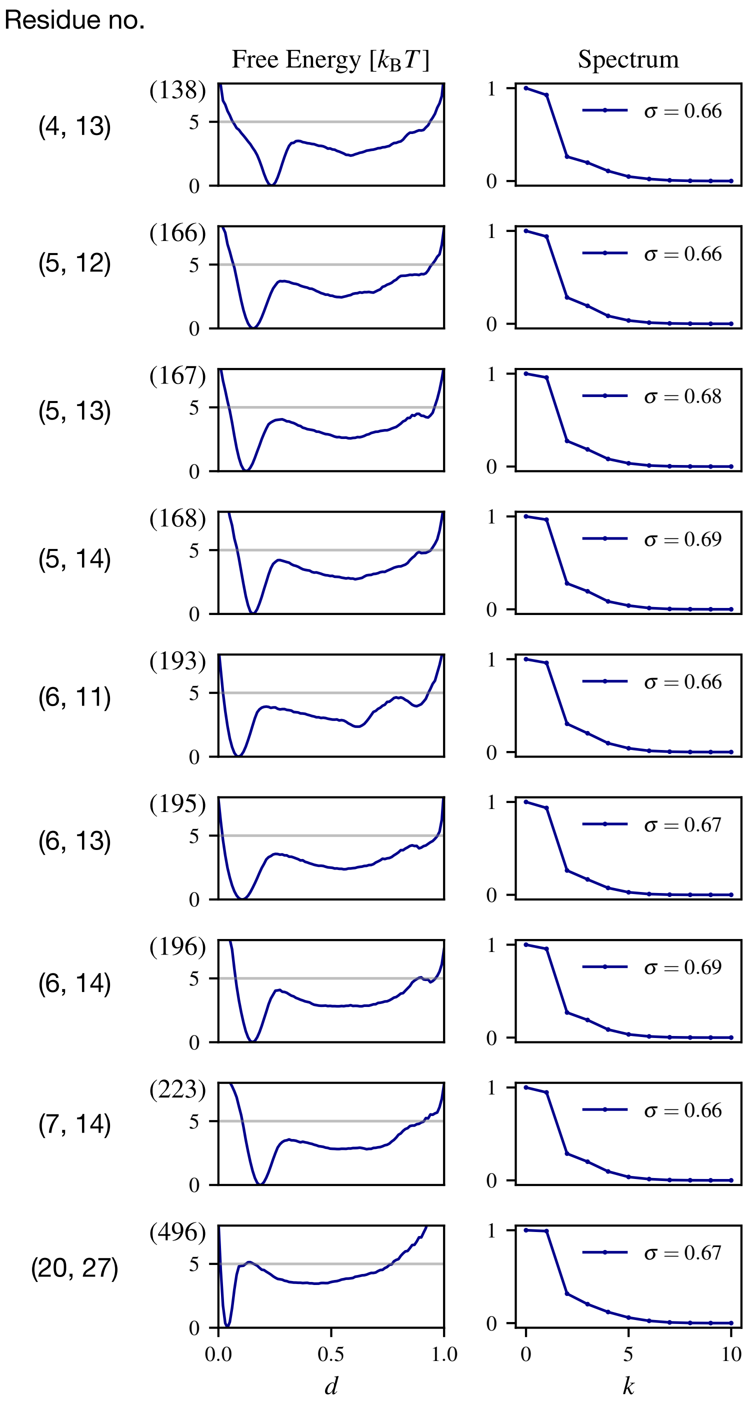

Figure S5: Free-energy profiles and eigenspectra for pairwise distances between Cα (indicated by residue numbers) of FiP35 for spectral gaps $\sigma > 0.65$.

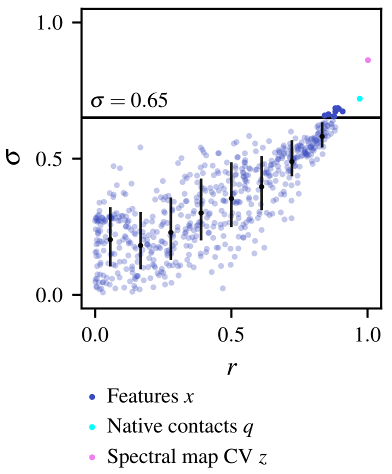

Figure S6: Scatter plot showing the features, fraction of native contacts, and slow CV in space spanned by the spectral gap σ and Pearson correlation coefficient with the slow CV r. Important features are shown above $\sigma > 0.65$.
